# Parents’ Positive Childhood Experiences: A Scoping Review

**DOI:** 10.1007/s42844-026-00219-1

**Published:** 2026-05-23

**Authors:** Emily Hoppe, Getachew Kassa, Zhiyuan Yu, Deborah Gross

**Affiliations:** 1 Johns Hopkins School of Nursing, Baltimore, MD USA; 2 University of Pennsylvania School of Nursing, Philadelphia, PA USA

**Keywords:** Parents, Positive childhood experiences, Psychological resilience, Intergenerational transmission, Scoping review

## Abstract

Positive childhood experiences (PCEs) are relational experiences occurring in childhood that confer a sense of safety and nurturance and are a powerful social determinant of health. Although these experiences may lay the foundation for later parenting practices, little is known about how parents’ PCEs influence the next generations’ childhood experiences and health outcomes. The purpose of this scoping review is to understand the extent and type of evidence available regarding parents’ PCEs and the potential impact of parents’ PCEs on intergenerational transmission of PCEs and protective factors for children’s health and psychosocial outcomes. Studies were eligible for inclusion if they were peer-reviewed and examined parents’ positive or benevolent childhood experiences. The literature search yielded 761 unique references in total, and ultimately 43 studies were included in the review. Parents’ PCEs were associated with better parent mental health and more positive parenting beliefs and attitudes, while outcomes were mixed for parenting behaviors and family and child health outcomes. Further research incorporating longitudinal and/or multiple data sources, as well as more direct examination of the proposed underlying mechanisms of PCEs on parenting and children’s health outcomes is needed. This is an area of research that has the potential to contribute to improving health across populations and merits significantly more and higher quality research to refine and deliver useful interventions for those parents most in need of support.

**Supplementary Information:**

The online version contains supplementary material available at https://doi.org/10.1007/s42844-026-00219-1.

## Key Findings

Parents’ positive childhood experiences (PCEs) were associated with better parent mental health and more positive parenting beliefs and attitudes, while outcomes were mixed for parenting behaviors and family and child health outcomes.

More than half of all research on parents’ PCEs has been published in the past five years, and this emerging field of study is characterized by use of validated measures, heterogenous samples, and focus on the developmentally sensitive peripartum and early childhood periods.

Studies of parents’ PCEs and the intergenerational transmission of PCEs and protective factors have not thoroughly examined explanatory models of underlying mechanisms of the benefits of parents’ PCEs for their children’s health and psychosocial outcomes.

**Supplementary Information:**

The online version contains supplementary material available at https://doi.org/10.1007/s42844-026-00219-1.

## Introduction

Positive childhood experiences (PCEs) are safe, nurturing relationships and environments that can support children’s healthy development and wellbeing (Hero et al., 2025). While the research on PCEs is emerging, studies have shown that they are associated with improved physical and mental health outcomes in children and adults (Crouch et al., 2024; Han et al., 2023). PCEs are also modifiable protective factors, representing opportunities at individual, family, and community levels to improve health outcomes. Current research on PCEs largely focuses on single-generation impacts; however, little is known about parents’ own positive early life experiences and how they influence their children’s childhood experiences and health outcomes. Understanding the impact of PCEs on parents and parenting may lead to greater understanding of how to influence intergenerational transmission of bio-psychosocial risk and protection.

The hallmark adverse childhood experiences (ACEs) study by Felitti, et al. (1998), led to recognition of the profound impact of childhood experiences on adult health and wellbeing (Kelly-Irving et al., 2013) and set off a wave of research into the impact of childhood adversity on adult health (Struck et al., 2021). However, PCEs were not investigated alongside ACEs until 2018 (Narayan, et al., 2018). After two decades of productive inquiry into the impacts of ACEs on health, in 2021, the American Academy of Pediatrics released a policy statement emphasizing the “biological necessity” of “safe, stable, nurturing relationships” for healthy childhood development and long-term health outcomes and calling for a “paradigm shift” in focus from adversity to relational health (Garner et al., 2021, p. 1). However, despite this re-orientation of research, policy, and clinical practice toward PCEs as predictors of health outcomes, this remains an under-developed area of science.

Despite evidence of PCEs’ positive effects on child development and adult health (Han et al., 2023), the mechanisms by which they provide these benefits, and the effects on later parenting practices, remain poorly understood (Narayan et al., 2021). For example, PCEs have been conceptualized as both “counter-ACEs,” or a protective buffer against the effects of ACEs, and as an independent, health-promoting factor (Crandall et al., 2019). In their systematic review, Han et al. (2023) found that the current science most supports that PCEs act as an independent health-promotive factor, however there are significant limitations in the current literature that leave unanswered the question of how PCEs impact parents, parenting, and family and child outcomes.

Several theoretical mechanisms by which PCEs affect parents and parenting have been described in the literature. These can be broadly categorized as resilience-based, attachment-based, or eco-developmental theories. Resilience-based theories conceptualize PCEs as protective resources that promote parental well-being, which may in turn support more adaptive parenting through reduced stress and better mental health (Chasson & Taubman – Ben-Ari, 2024; Morris et al., 2021). Attachment-based theories, including angels in the nursery theory, view PCEs as formative relational templates established early in life that shape caregiving expectations and behaviors carried into adulthood (Narayan et al., 2020). Finally, eco-developmental theories focus on how the environment provides either risk or protective exposures to both the parent and child across their lifespans (Boyce et al., 2021; Heberle et al., 2014).

Although many ACEs and PCEs are directly influenced by parental health and behavior, research examining the impact of PCEs on parents and their children is limited. There has been more research on the intergenerational impact of ACEs than on the intergenerational impact of PCEs (Narayan et al., 2021). Several recent studies have highlighted the relationship between parental ACEs and child behavioral concerns (Schickedanz et al., 2018; Zhang et al., 2023). While several systematic reviews have been published on the intergenerational impacts of ACEs (Racine et al., 2023; Rowell & Neal-Barnett, 2022; Zhang et al., 2023), according to the available literature, there are no known published systematic reviews on the topic of intergenerational PCEs. A systematic review by Han et al. (2023), examined PCEs’ impact on adult outcomes in the context of adversity. A scoping review by Raghunathan et al. (2024) focused on how PCEs have been operationalized and measured in the literature. The present scoping review differs from these reviews in its focus on mapping the evidence regarding parents’ PCEs, a sub-population of particular interest for understanding the intergenerational transmission of protection and risk, and in which PCEs may have distinct prevalence, characteristics, contexts, and functions. Furthermore, to our knowledge this is the first systematic review to focus on parental PCEs’ influence on child and family outcomes.

### Purpose and Aims

The purpose of this scoping review was to understand the extent and type of evidence available regarding parents’ PCEs and their potential impact on the intergenerational transmission of PCEs and potential protective factors for children’s health and psychosocial outcomes. This scoping review is needed to guide future research directions as well as inform policy and clinical interventions. The specific aims of the review were:

Aim 1: Understand the state of the science regarding parents’ PCEs, including populations represented and designs, methods, and theoretical models used to describe relationships between PCEs and parent, family, and child outcomes.

Aim 2: Understand how parents’ PCEs relate to parental beliefs, attitudes, behaviors, and mental health and well-being, and their children’s health outcomes.

## Methods

The search methods were guided by the Preferred Reporting Items for Systematic reviews and Meta-Analyses extension for Scoping Reviews (PRISMA-ScR; Tricco et al., 2018). A scoping review approach was indicated due to the emerging nature of the field of PCEs research and the need for a clear understanding of the state of the science and the current gaps in knowledge regarding parents’ PCEs. The protocol for this scoping review was registered and is publicly available through the Open Science Framework (Hoppe et al., 2025). A preliminary search of MEDLINE, the Cochrane Database of Systematic Reviews, *JBI Evidence Synthesis*, and the Open Science Framework database was conducted, and no published or in progress systematic reviews on the topic of parents’ PCEs were identified.

### Eligibility Criteria

Studies were eligible for inclusion if they were peer-reviewed and examined parents’ PCEs, including benevolent childhood experiences, safe, stable, and nurturing relationships, and positive or safe experiences of community. The concept of “parent” was broadly defined to encompass any primary caregiver of children, non-residential parents and caregivers, or pregnant people expecting a child. Articles were not excluded by date, geographic location, or setting. Experimental and quasi-experimental study designs, analytical observational studies including prospective and retrospective cohort studies, case-control studies, case series, mixed methods, and descriptive and analytical cross-sectional studies were included for consideration. Qualitative studies were also included. Case studies, conference presentations, abstracts, dissertations, protocol papers, opinion articles, and literature reviews were excluded. Non-English-language studies were excluded due to authors’ language proficiency.

### Information Sources and Search

Six databases were searched: PubMed, CINAHL, PsycInfo, Embase, Scopus, and Sociological Abstracts. A health informationist assisted with adapting search terms for each database. Sample search terms included: “positive childhood experiences,” “benevolent childhood experiences,” “counter ACEs,” “safe stable nurturing relationships,” “advantageous childhood experiences,” and “counter ace.” Complete information about search terms for each database is included in Appendix A. Because positive childhood experiences had not been indexed as a Medical Subject Headings term, we were concerned that articles would not be systematically indexed. Therefore, we did not narrow the search with population-based search terms to ensure that eligible studies would not be inadvertently excluded. The reference lists of included articles were screened for additional studies. The search was initially conducted on March 27, 2023, and then repeated using the same search terms on April 28, 2025. See Supplemental materials for additional search information.

### Selection of Evidence

**Fig. 1:**
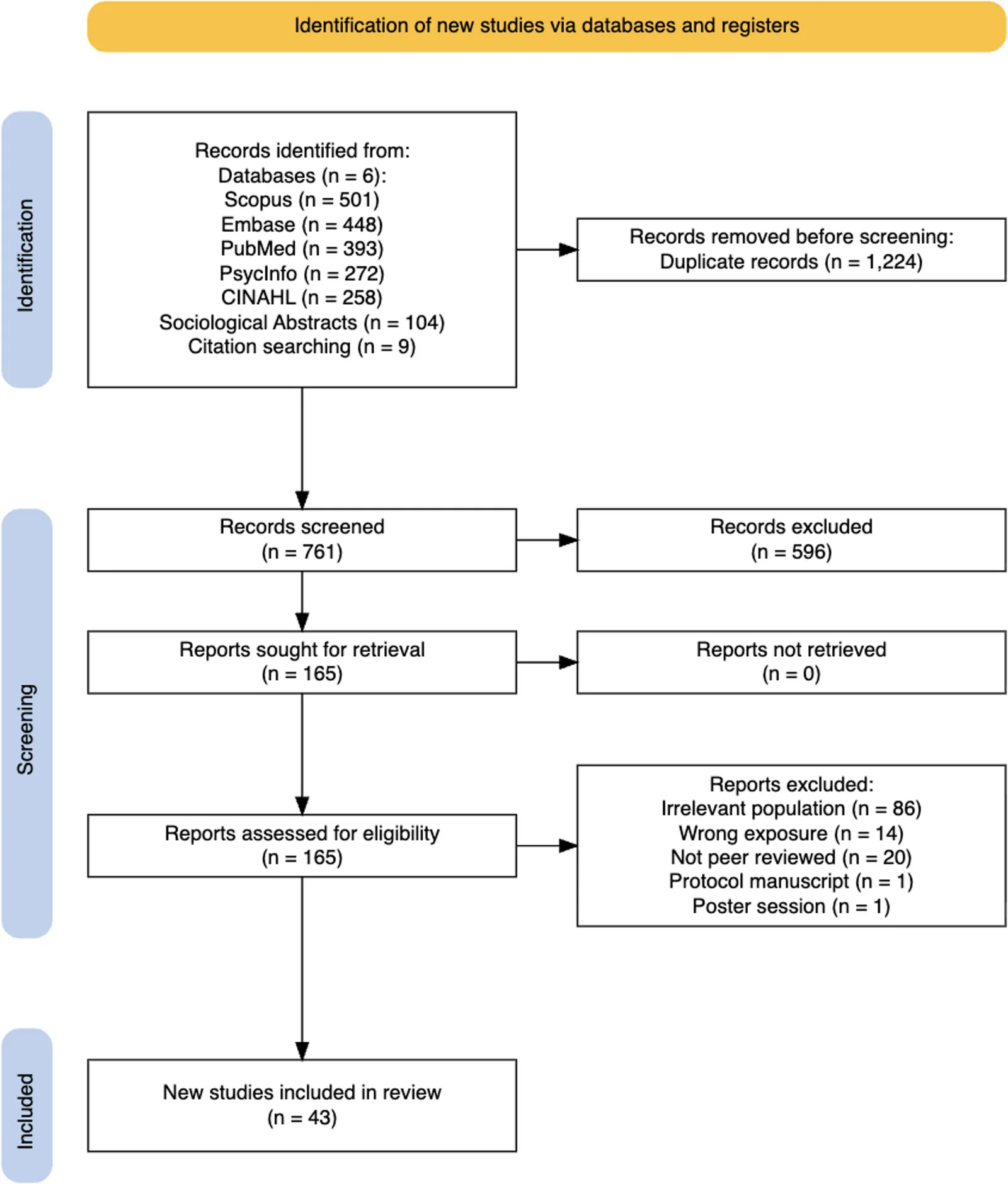
PRISMA flow diagram

Following the search, all identified citations were collated and uploaded into Covidence, and duplicates were removed (www.covidence.org). Title/abstract screening was conducted independently by two authors (GK and EH) for assessment against the inclusion/exclusion criteria. Potentially relevant full texts were retrieved and reviewed in detail independently by two reviewers to determine whether they met inclusion criteria. Discrepancies in assessment arising at any stage were resolved through discussion and consensus between the reviewers. When consensus could not be reached or additional insight was needed, senior author provided additional guidance. The search and screening process is detailed in Fig. 1 (Haddaway et al., 2022).

### Data Extraction

Data extraction was facilitated by a tool developed by the authors using the web-based survey software Qualtrics (https://www.qualtrics.com). The tool included items for extraction, including information about the study sample and setting, study design, methods, and findings. Two authors independently extracted studies and compared results, resolving discrepancies through consensus.

### Critical Appraisal

Critical appraisal is not a required component of scoping reviews (Tricco et al., 2018). However, to aid assessment of the state of the science, a brief, 4-item appraisal tool was developed to assess the quality of studies, including items on ethics approval, use of a validated PCEs measure, outcomes measurement, and sound statistical analysis. The results of the critical appraisal were used as background to inform the authors’ synthesis and interpretation of the articles as a whole body of knowledge. Individual articles were not excluded on the basis of appraisal.

## Results

**Fig. 2:**
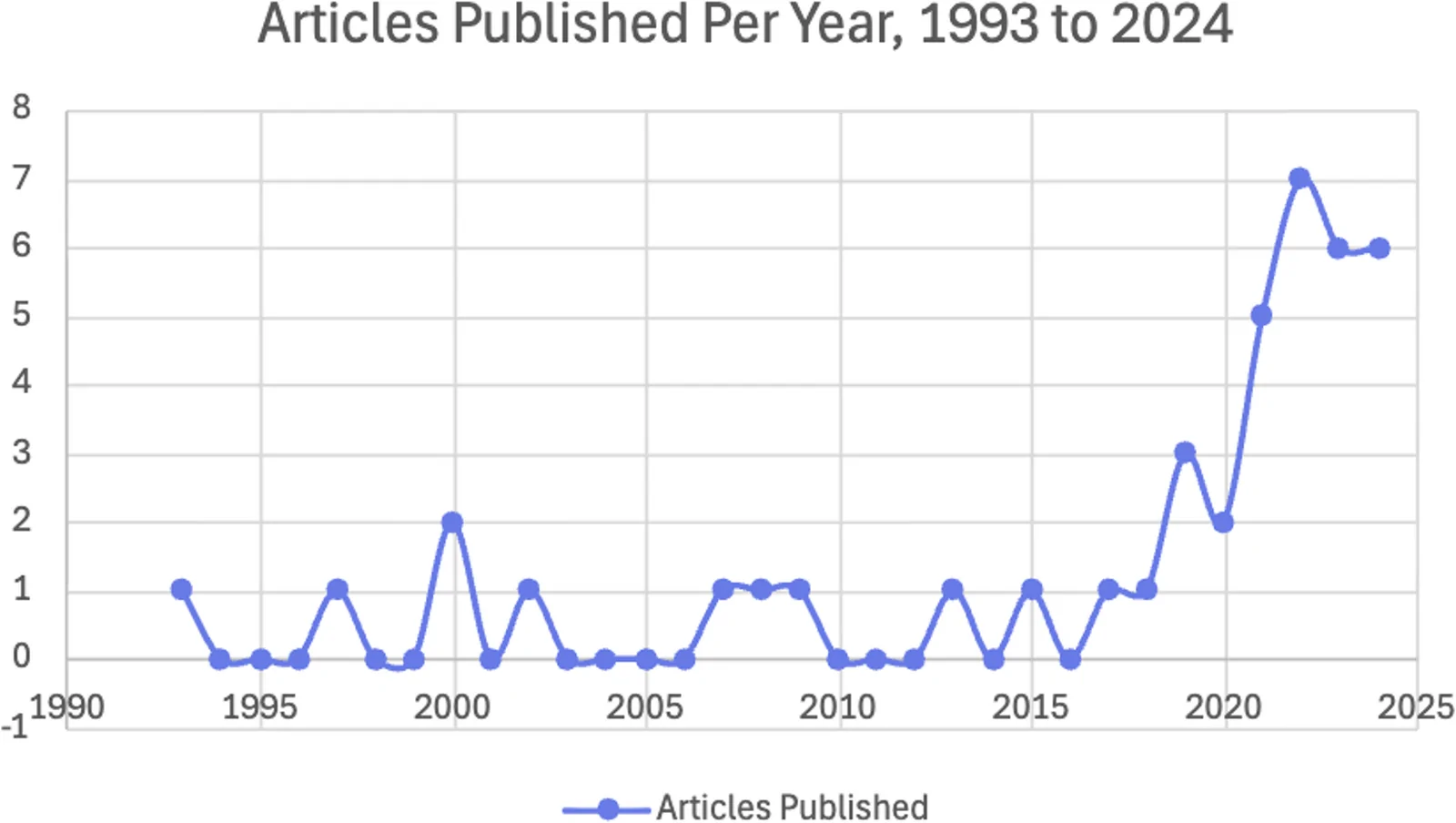
Number of articles published per year on parents’ PCEs, 1993-2024

 The literature search yielded 1,885 references in total – 1,167 in March 2023, and 718 when the search was repeated in April 2025 (for only the dates since the previous search). After duplicates were removed, 760 studies were screened using title and abstract review from which 164 were sent to full text review, and ultimately 43 studies were included (see Fig. 1). Reasons for exclusion after full text review were irrelevant population (e.g. not specifically parents; *n* = 86), wrong exposure (e.g., positive experiences in adulthood instead of childhood; *n* = 14), not peer reviewed (*n* = 20), or wrong reference type (one protocol paper and one poster abstract). Quantitative study characteristics and findings, and qualitative study details for the 43 included studies are presented in Tables 1, 2 and 4 respectively. Studies examining parents’ PCEs were rare until 2019, at which point the number of articles increased, but have not exceeded seven published studies per year through 2024 (see Fig. 2).

**Table 1:** Summary of study characteristics (*N* = 43)

1^st^ Author (Year)	Country	Study Population	Data Source	Parent Analytic Sample Size	Design	Parent Race/ Ethnicity %	Parent sex/ gender %	Parent Age Mean Years (Range)	Child Age Mean (Range or SD)	Mostly low SES?	Urban or Rural?
Bailes (2024)	USA	Pregnant people	Authors collected	298	CSS within LC	73 W, 8 B/AA, 4 MR/O, 2 A, 1 NHPI, 7 H.	NR	38.8 (19–45)	NA - prenatal	NR	Urban
Baptista (2024)	Portugal	Mothers of infants	Authors collected	95	CSS	NR	100 F	32.1 (20–45)	10 mos (range 6–15, sd = 3.3)	NR	NR
Barnert (2023)	USA	Parent-child dyads	PSID - 2013	1,854 dyads	LC	87 W, 10 B/AA, 4 L, 2 AAPI	NR	NR	38.5 yrs (sd = 10.9)	NR	Both
Bartlett (2015)	USA	Young mothers in home-visiting program	Authors collected	447	3-wave, MM study with RCT	35 W, 31 H, 20 B/AA, 10 MR, 4 O	100 F	18.73	12 mos (range 1.8–29.0.8.0 mos)	Yes	Both
Beaton (2007)	USA	Couples expecting first child	Authors collected	131 couples	3-wave study with RCT	81 W, 16 interracial couples, 2 B/AA, 1 AsA	50 F, 50 M	Men: 32.6 Women: 30.7	6–12 mos	No	NR
Blackwell (2024)	USA	Parents of 1–5 year old	Authors collected	1,016	CSS	NR	68 F, 32 M	Median = 35	Median = 3.7 yrs (range = 1–5)	No	NR
Cárdenas (2022)	USA	Pregnant women	Authors collected	208	LC	82 W, 10 B/AA, 9 H, 5 MR/other, 2 AsA, 1 NHPI	100 F	30.3 (sd = 5.5)	1–6 mos	No	NR
Chasson (2022)	Israel	Mothers of newborns	Authors collected	T1: 715 T2: 392	LC	Race/ethnicity NR; 98 Jewish	100 F	NR	T2: 8.8 mos (sd =1.5)	NR	NR
Chasson (2024)	Israel	Postpartum women	Authors collected	T1: 715 T2: 392	LC	Jewish 98.7, Muslim 0.5, Christian 0.3, O 0.5	100 F	31.4 (20–45)	T2: 6–10 mos	No	NR
Chung (2008)	USA	Pregnant women	Authors collected	1,476	LC	71 B/AA, 17 L, 9 W, 3 O	100 F	24 (sd = 6)	T2: 11 mos	Yes	Urban
Clark (2023)	USA	Pregnant women	Authors collected	292	CSS	48 W, 15 B/AA, 7 AI/AN, 5.1 AsA, 6 MR, 1 NHPI; 27 H	100 F	30 (sd = 5.8)	NA - prenatal	No	Urban
Crouch (2023)	USA & Canada	Lactating individuals with child 0–5 years old	Authors collected	617	CSS	88 W, AsA 7, B/AA 2, NA 2, 1 PI/AN	100 F	33.7 (range = 26–49)	5 yrs or younger	NR	NR
Daines (2021)	USA	Parents	Authors collected	1,030	CSS	61 W, 39 NR	46.5 M, 53.5 F	40.4 (sd = 17.3)	NR	NR	NR
Geng (2021)	China	Parents	Authors collected	7,218	CSS	NR	33.1 M, 66.9 F	38.1 (sd = 7.8)	School-aged	No	Both
Geng (2021a)	China	Parents of school-aged children	Authors collected	7,245	CSS	NR	32.7 M, 67.3 F	38.1 (sd = 7.8)	School-aged	No	Both
Gissandaner (2024)	USA	Parent-child dyads preschool-aged	Authors collected	125	CSS	57 W, 26 H, 8.0 B/AA, 6 AsA, 2 BR, 1 NA	13.6 M, 86.4 F	33.3 (sd = 6.8)	4 yrs (sd = 0.8)	NR	Urban
Goodyear (2002)	USA	Pregnant or recently pregnant Latina young women	Authors collected	493	CSS	100 L	100 F	16.8 (sd = 1.3)	Pregnant mothers and infants up to 3 mos	NR	Urban
Greif (2000)	USA	African American mothers of UMBC Meyerhoff program scholars	Authors collected	38	Qualitative	100 B/AA	100 F	47	College-aged and older	NR	NR
Herrenkohl (2013)	USA	Parents	Authors collected - Lehigh Longitudinal Study	268	LC	78 W, 11 MR, 9 B/AA, and a small number were AsA (*n*=1), AI/AN (*n*=2) and NH/PI (*n*=2)	51.5 M, 48.5 F	36.4 (range = 32–41)	NR	Yes	Mostly suburban
Johnson (2022)	Canada	Parent-child dyads aged 5–18 yrs	Authors collected	547	CSS	74 W	29.9 M, 70.1 F	41 (sd = 6)	5–18 yrs	NR	NR
Johnson (2023)	USA, UK, Canada, Australia	Parents of at least 2 children 5–18 yrs	Authors collected	548	LC	74 W	30 M, 70 F	41 (sd = 6)	5–18 yrs	No	NR
King (2023)	USA	Mothers of a biological child preschool aged	Authors collected	97	LC	71 W, 22 AsA, 2 AI/AN, 5 O, 4 B/AA	100 F	37.2 (sd = 4.6)	4.2 yrs (sd = 0.9)	No	NR
McConnell (2022)	Canada	Adults receiving support for individuals with Intellectual Disability	Authors collected	91	CSS	NR	9.9 M, 90.1 F	38.9 (sd = 10.7)	NR	NR	NR
Merrick (2019)	USA	Parents of children aged 4–6 living at an emergency homeless shelter	Authors collected	50	CSS	66 B/AA, 12 W, 12 AI, 10 BR/O	12 M, 88 F	32.5 (sd = 9.3)	5.4 yrs (sd = 0.8)	Yes	Urban
Merrick (2020)	USA	Pregnant women	Authors collected	101	CSS	37 L, 22 B/AA, 20 W, 7 AsA/PI, 13 BR/MR, 1 AI	100 F	29.1 (sd = 6.6)	NA – prenatal	Yes	Urban
Merrick (2024)	USA	Pregnant individuals	Authors collected	T1: 175 T2: 121	LC	39 W, 26 L, 17 B/AA, 12 BR/MR, 7 O	NR	28.1 (sd = 5.68)	T1: prenatal T2: 3–4 mos	Yes	Urban
Morris (2021)	USA	English-speaking, adult parents	Authors collected	109	CSS	58 W, 25 NA, 13 B/AA, 4 L	34.9 M, 65.1 F	38.1 (25–50)	NR	Yes	Urban
Narayan (2017)	USA	Mothers with exposure to foster care in childhood	Authors collected	54	CSS - MM	59 B/AA, 13 W, 6 L, 22 BR/MR	100 F	32.8 (sd = 8.9)	NR	Yes	NR
Narayan (2018)	USA	Pregnant women	Authors collected	101	LC	37 L, 22 AA, 20 W, 13 BR/MR, 7 AsA/PI, 1 AI	100 F	29.1 (sd = 6.6)	NR	Yes	Urban
Narayan (2019)	USA	Mothers of young children exposed to a traumatic event	Authors collected	185	CSS - MM	55 L, 18 W, 10 AA, 9 BR/MR, 5 A, 3 O	100 F	30.7 (17–46)	43 mos (sd = 16 mos)	Yes	Urban
Naryan (2019a)	USA	Couples experiencing pregnancy	Authors collected	173	CSS – MM	Women: 35 W, 21 B/AA, 20 L, 16 BR/MR, 8 O Men: 31 W, 26 B/AA, 22 BR/MR, 17 L, 4 O	41.6 M, 58.4 F	Women: 27.6 (sd = 5.7); Men: 29.4 (sd = 7.8)	NR	Yes	Urban
Narayan (2020)	USA	Pregnant women	Authors collected	T1: 101 T2: 77	LC – MM	37 L, 22 AA, 20 W, 13 BR/MR, 8 O	100 F	29.1 (18–44, sd = 6.56)	3–4 mos	Yes	Urban
Nevarez-Brewster (2022)	USA	Pregnant women	Authors collected	164	LC	51 W, 26 L, 12 B/AA, 6 AsA/A, 3 MR, 1 AI/AN, 1 NH/PI	100 F	30 (sd = 5.4)	NA - prenatal	No	Urban
Onyskiw (1997)	Canada	Couples with biological newborn child	Authors collected	66	CSS nested in LC	99 W	50 M, 50 F	Women: 29.8 (sd = 5.2) Men: 32.3 (sd = 5.7)	12 mos	No	Urban
Prime (2022)	USA, UK, Canada, Australia	Caregivers of children aged 5–18 years	Authors collected	549	CSS	73 W, 27 NR	32 M, 68 F	NR	Younger child: 9.6 (sd = 3.2); Older child: 11.8 (sd = 3.32)	No	NR
Reese (2022)	USA	Couples with child aged 3–13 years	Authors collected	964	CSS	73 W	50 M, 50 F	Women: 35.6; Men: 38.9	9.75 yrs (range: 3–13)	NR	NR
Siddiqui (2000)	Sweden	Mothers	Authors collected	171	CSS	100 “ethnically Swedish”	100 F	30 (sd = 4.6)	NR	NR	Both
Simons (1993)	USA	Parents of 7th graders	Authors collected – Iowa Youth and Families Project	902	LC	100 W	50 M, 50 F	NR	NR	No	Rural
Suzuki (2009)	USA & Japan	Parents of preschool-aged children	Authors collected	235	CSS	USA: 89 W, 4 L, 3 AsA, 3 O; Japan: NR	100 F	USA: 38.1 (sd = 4.2); Japan: 35.6 (sd = 3.8)	USA: 55 mos (sd = 4); Japan: 68 mos (sd = 3.5)	No	Urban
Tadjine (2025)	Ireland	First-time, single mothers	Authors collected	3	Qualitative	NR	100 F	“early thirties”	NR	Yes	NR
Yu (2025)	USA	Parents of a 2–8-year‐old child enrolled in a Baltimore City public school	Authors collected	230	Quasi-experimental	67 B/AA, 9 W, 5 MR, 22 L	5 M, 95 F	35 (sd = 9.5)	2–8 yrs	Yes	Urban
Zhan (2021)	China	Parents of school-aged children	Authors collected	6,929	CSS	NR	32.9 M, 67.1 F	38.0 (sd = 7.8)	School-aged children	NR	Both
Zhu (2023)	China	Parents of school-aged children	Authors collected - Anhui Family Cohort Study	2,587	CSS	NR	100 F	34.4 (sd = 4.9)	64 mos (sd = 10.3)	NR	NR

*H *hispanic, *L *latina/o/x, *AA* African American, *B* black, *AAPI* Asian American/Pacific Islander, AsA Asian American, *A* Asian, *MR* multiracial/ethnic, *BR* biracial, *NHPI *native Hawaiian/Pacific Islander, *O* other, *AI/AN* American Indian or Alaska Native, *NA* Native American, *F* female, Women, Mothers, or Girls, *M* male, men, or boys *NR* not reported, *MM* mixed methods, *RCT* randomized control trial, *CSS* cross-sectional survey, *LC* longitudinal cohort, *PSID* panel study of income dynamics

Articles variously reported “mothers,” “women,” and “females,” which have been collapsed into “F” in this table, similarly “fathers,” “men,” and “males” are categorized here as M. There were no articles that reported non-binary or other genders. In articles that included “pregnant individuals” without reporting gender, NR is used in this table

**Table 2:** Summary of quantitative article findings by outcome (*n* = 41)

Authors (Year)	Outcome of Interest	PCEs Measure Used	Mean Score (sd) of Parental PCEs	Prevalence of Parental PCEs	Statistically Significant PCEs Effect Sizes or Mediation/Moderation Effects	Summary of PCEs-Related Findings
Parent Outcomes – Mental Health (*n* = 15)						
Chasson (2024)	Personal post-traumatic growth	BCEs scale (Narayan, 2018)	NR	NR	Indirect effect of BCEs on outcome via self-compassion: β = 0.03, SE = 0.014, via social support: β = 0.01, SE = 0.008, via psychological distress and self-compassion: β = 0.006, SE = 0.003, via psychological distress and social support: β = 0.004, SE = 0.002	Women who reported greater BCEs reported higher personal growth in Phase 2 through indirect effects of self-compassion, psychological distress, and social support.
Cardenas (2022)	Depressive symptoms	BCEs (Narayan 2018)	9.3/10 (1.34)	NR	Bivariate correlations: −0.21 antepartum depressive symptoms −0.26 depressive symptoms at newborn visit −0.27 depressive symptoms 6 months postpartum	BCEs were associated with lower depressive symptoms collapsed across peripartum period. BCEs predicted postpartum depressive symptoms over and above antepartum depressive symptoms.
Chung (2008)	Depressive symptoms	Author-generated instrument	NR	Total no. of positive influences in childhood (PICs) 0: 4% 1: 12% 2: 28% 3: 26% 4: 30%	0.43 and 0.42 aOR for having 3 PICs or 4 PICs and odds of depressive symptoms.	Increased PICs were associated with lower rates of depressive symptoms. Maternal relationship but not paternal relationship moderated the effect of sexual abuse.
Clark (2023)	Psychopathology	BCEs (Narayan 2018)	8.6/10 (1.93)	NR	General psychopathology: ß = −0.268 Thought problems: ß = −0.284 Internalizing symptoms, ß = −0.197 Detachment ß = −0.249 Disinhibited externalizing behavior: ß = −0.352 Antagonistic externalizing behavior: ß = −0.175	BCEs are negatively associated with broad psychopathology during pregnancy, even after accounting for contemporaneous traumatic life events. Links between childhood BCEs and latent psychopathology constructs were strongest for forms of psychopathology closer to the top of the Hierarchical Taxonomy of Psychopathology.
Geng (2021)	PTSD symptoms	BCEs (Narayan 2018)	NR	NR	−0.03 association via linear regression	Positive childhood experiences were negatively related to PTSD symptoms.
Johnson (2022)	Parent mental health, child mental health, family functioning	BCEs scale (Narayan, 2018)	NR	BCEs Count by latent class: Low ACEs/High BCEs (49.4%): 6.45; Moderate ACEs/High BCEs (26.7%): 6.04; Moderate ACEs/Low BCEs (12.8%): 3.04; High ACEs/Moderate BCEs (11.2%): 4.25	Significantly higher parental anxiety, psychological distress, and posttraumatic stress were observed in the Moderate-ACEs/High-BCE, Moderate-ACEs/Low-BCEs, and High ACEs/Moderate-BCEs groups compared to the Low-ACEs/High BCEs group.	No significant associations were observed between the latent classes and either child positive coping or parenting quality. Parent mental health problems were observed less in the latent class with low ACEs/high BCEs compared with the other groups.
Johnson (2023)	Parental mental health during Covid-19 pandemic	BCEs scale (Narayan 2018)	5.7/7 (1.57)	BCE 1: 92% BCE 2: 96% BCE 3: 70% BCE 4: 73% BCE 5: 79% BCE 6: 81% BCE 7: 76%	0.71 OR for psychological distress	No observed interactions between BCEs and ACEs, or BCEs and COVID stress, for any mental health outcome. BCEs are associated with lowering this risk of mental health concerns.
King (2023)	Maternal mental health, child behavior problems	BCEs scale (Narayan 2018)	9.4/10 (1.13)	NR	NR	Mothers who reported having fewer positive experiences in childhood reported being exposed to a higher number of adverse experiences in childhood.
Merrick (2019)	Parent psychological distress and sociodemographic risk	BCEs (Narayan 2018)	7.6/10 (2.23)	BCE 1: 94%, BCE 2: 86%, BCE 3: 76%, BCE 4: 68%, BCE 5: 86%, BCE 6: 66%, BCE 7: 68%, BCE 8: 80%, BCE 9: 64%, BCE 10: 68%	−0.35 BCEs association with psychological distress	BCEs significantly predicted psychological distress. BCEs were not associated with parenting stress. Notably, homeless parents endorsed the BCEs item about the presence of a predictive home routine significantly less often than low-income pregnant women (68% vs. 81%).
Merrick (2020)	Maternal depression and PTSD symptoms, risky reproductive planning, stressful life events (SLEs)	BCEs (Narayan 2018)	Early childhood 4.7 (2.83); Middle childhood 6.5 (2.73); Adolescence 7.02 (2.50)	Onset of BCEs (≥6 BCEs present): 43% early childhood, 28% middle childhood, 12% adolescence.	−0.25 association with risky reproductive planning; −0.21 stressful life events	BCEs in adolescence did not significantly predict prenatal depression symptoms. BCEs at any developmental period did not predict prenatal PTSD symptoms. Higher BCEs in early childhood and adolescence predicted less risky reproductive planning. Higher BCEs at each developmental period predicted prenatal SLEs.
Merrick (2024)	Pre- and post-natal depression and PTSD symptoms	BCEs scale (Narayan, 2018)	8.2/10 (1.66)	25.7% positively endorsed all 10 BCEs	Prenatal depression *r* = −.19; Prenatal PTSD *r* = −.24; Postnatal depression *r* = −.21; Postnatal PTSD *r* = −.23; Sociodemographic stress *r* = −.26.	Higher BCEs were significantly associated with lower levels of prenatal and postnatal mental health problems and lower levels of prenatal sociodemographic stress. They were not significantly associated with higher levels of prenatal or postnatal social support.
Narayan (2017)	Parental PTSD symptoms	Angels in the Nursery Interview	3.1/5 (1.29)	High Angels, Low Ghosts: 50% High Angels, High Ghosts: 33% Low Angels, High Ghosts:15%	The simple slope of adulthood PTSD symptoms on childhood maltreatment was significant when angel memories were low (1 SD below mean) but not when angel memories were moderate (at the mean) or high (1 SD above mean).	Higher angel memories may protect mothers who were maltreated from experiencing PTSD symptoms.
Narayan (2018)	Prenatal depression and PTSD symptoms, perceived stress	BCEs (Narayan 2018)	7.8/10 (2.14)	Mean levels of BCEs did not significantly differ by language of administration, place of birth, or race.	−0.24 to PTSD; −0.23 to prenatal SLEs	After accounting for ACEs, BCEs scores significantly predicted prenatal PTSD symptoms and SLEs, but not depression symptoms and perceived stress. Higher BCEs seemed to be beneficial for women with heightened childhood adversity.
Narayan (2019a)	Parent depression and PTSD symptoms	BCEs (Narayan 2018)	Women: 8.1/10 (1.7) Men: 8.0/10 (1.6)	WOMEN: BCE 1: 96%, BCE 2: 90%, BCE 3: 72%, BCE 4: 73%, BCE 5: 83%, BCE 6: 73%, BCE 7: 75%, BCE 8: 89%, BCE 9: 68%, BCE 10: 76% MEN: BCE 1: 89%, BCE 2: 82%, BCE 3: 74%, BCE 4: 67%, BCE 5: 86%, BCE 6: 74%, BCE 7: 65% BCE 8: 93%, BCE 9: 82%, BCE 10: 67%	MEN: −0.32 depression symptoms, −0.31 PTSD symptoms; WOMEN: −0.27 PTSD	Women’s BCEs and ACEs independently affected their level of PTSD symptoms during pregnancy. For fathers-to-be, higher levels of ACEs, but not lower levels of their BCEs, predicted higher levels of PTSD and depression symptoms during the pregnancy period.
Zhan (2021)	Parent depression and PTSD symptoms, parent prosocial behaviors	BCEs scale (Narayan 2018)	8.6/10 (1.73)	Participants who resided in urban settings reported higher levels of PCEs than those in rural settings. ANOVA indicated that individuals with higher levels of education reported more PCEs.	β = −0.06 PTSD symptoms; β = −0.10 depression symptoms; β = 0.15 prosocial behaviors	PCEs were negatively correlated with symptoms of PTSD and depression, while positively correlated with prosocial behaviors.
Parent Outcomes – Parenting Beliefs, Attitudes, and Behaviors (*n* = 13)						
Bailes (2024)	Prenatal mental representation of relationship with child	BCEs scale (Narayan, 2018)	9.3/10 (1.37)	NR	No statistically significant associations with representation classifications.	PCEs did not appear to buffer the potential negative associations between stressful life experiences and prenatal representations.
Baptista (2024)	Maternal emotional responses to infant crying	BCEs scale (Narayan, 2018)	8.7/10 (1.63)	NR	No significant associations were found between BCEs and maternal responses to infant crying.	BCEs did not predict child-oriented emotions, which may be due to the high mean BCE score in our study.
Beaton (2007)	Attitudes about fatherhood	Family of Origin Questionnaire (FOQ)	NR	NR	Association between fathers’ closeness with their fathers and fathers’ attitudes about fatherhood: T2: β = 1.93 T3: β = 1.74	In a parenting classes intervention study, fathers’ closeness with their fathers during childhood had a significant linear and curvilinear association with their attitudes about fatherhood at 6 and 12 months after the birth of a first child.
Chasson (2022)	Parent role satisfaction	BCEs scale (Narayan, 2018)	8.35/10 (sd 1.70)	NR	Bivariate correlation: maternal self-efficacy *r* =.13, maternal role satisfaction *r* =.19	BCEs were associated with higher maternal self-efficacy and role satisfaction, serially mediated by self-compassion and disintegrative experiences.
Herrenkohl (2013)	Physical discipline and harsh parenting	Author-generated mother, father, and sibling care scales adapted from Midlife in the United States national survey	Mother care 3.23/4 (sd 0.80); Father care 3.10/4 (sd 0.78); Sibling care 2.99/4 (sd 0.77)	NR	Caring relationship with father is associated with lower odds of harsh physical discipline by Generation 2 parents (β = −0.24)	None of the SSNR variables mitigated the effect of Generation 1 harsh physical discipline on similar practices of Generation 2 parents. Paternal care in childhood directly associated with lower use of harsh physical discipline.
McConnell (2022)	Parenting role satisfaction, perceived social support	Parental Bonding Instrument (PBI)	Maternal care 2.2/4 (sd 0.71) Paternal care 2.2/5 (sd 0.68)	NR	Paternal care in childhood was positively associated with perceived social support (*r* =.24), parenting role satisfaction (*r* =.23) and emotional warmth (*r* =.30).	Parental role satisfaction, perceived social support, and warmth were statistically significantly positively associated with parents’ paternal, but not maternal, care in childhood.
Morris (2021)	Nurturing and harsh parenting attitudes	10-item PACEs survey (Morris 2018)	7.5/10 (sd 2.25)	NR	PACEs were positively associated with nurturing parenting attitudes (β = 0.33) and not statistically significantly associated with harsh parenting.	Moderation analyses indicated that the association between ACEs and harsh parenting attitudes was conditional upon the level of PACEs. When PACE scores were low (M – 1 SD), but not when PACE scores were average or high (M + 1 SD), ACEs were associated with harsh parenting attitudes, suggesting a buffering effect of PACEs on negative parenting attitudes.
Narayan (2020)	Angels and ghosts in the nursery	BCEs scale (Narayan 2018)	7.8/10 (sd 2.14)	NR	BCEs association with prenatal angel memories β = 0.34. There was a significant indirect effect of higher BCEs on higher postnatal angel memories through higher prenatal angel memories β = 0.14.	Higher levels of BCEs from mothers’ families of origin indirectly predicted higher levels of mothers’ angel memories at 3 to 4 months postnatal, linking childhood experiences in the first generation to the potential for more positive caregiving in the second generation.
Onyskiw (1997)	Quality of parenting interactions	Parental Acceptance-Rejection Questionnaire (Rohner et al., 2005) Min. possible 60, max. possible 240. Higher = more rejection	Women: 104 (34.7) Men: 97 (29.9)	NR	For fathers: β = −2.63 for quality parenting interactions, β = 2.76 interaction of BCEs x marital support predicting quality of parenting interactions	There was no relationship between maternal childhood experiences and the quality of parenting interactions. Fathers who recalled less positive childhood experiences but had higher levels of marital support were predicted to have more optimal parenting interactions.
Siddiqui (2010)	Prenatal attachment	Own Memories of Child Rearing (EMBU)	NR	NR	ρ = 0.20 fantasy subscale; ρ = 0.22 sharing pleasure subscale	Women who reported more emotional warmth from their mothers were found to be keener on sharing the pleasure about their pregnancy and expressed more fantasy.
Simons (1993)	Supportive parenting and harsh discipline	The self-report measure consisted of a 9-item Supportive Parenting Scale (Simons et al., 1992)	NR	NR	β = 0.13 supportive parenting; β = 0.13 satisfaction with child	Grandparents’ supportive parenting is associated with parents’ satisfaction with child and supportive parenting.
Suzuki (2009)	Parenting self-efficacy	Childhood parental support scale (authors developed)	USA: 4.2 on scale of 1–6 (1.1) Japan: 4.0 (1.1)	NR	0.26 correlation with parenting self-efficacy; β = 0.152 multivariable regression on parenting self-efficacy	Mothers with higher parenting self-efficacy reported receiving more positive childhood parental support.
Yu (2025)	Engagement in a school-based parenting program	PCEs measure (Bethell 2019)	3.7/7 for virtual group participant; 4.1/7 for in-person group participants	0–2 PCEs: 33.5%; 3–5 PCEs: 32.6%; 6–7 PCEs: 33.9%	No statistically significant results.	In a quasi-experimental study of a parenting skills group intervention, frequencies of parent-reported ACEs and PCEs were unrelated to parenting program session attendance or quality of parent engagement in the program sessions.
Parent Outcomes – Other (*n* = 4)						
Crouch (2023)	Parent marijuana use	PCEs measure (Bethell 2019)	NR	0 PCEs: 3.4%; 1–2 PCEs: 10.4%; 3–4 PCEs: 19.1%; 5+ PCEs: 67.0%	NR	There was no evidence to suggest moderation of the relationship between ACEs and marijuana use by PCEs.
Geng (2021a)	Parent insomnia	BCEs (Narayan 2018)	8.6/10 (1.80)	NR	Multivariable regression to predict insomnia: β = −0.09 Mediation pathways: PCEs were associated with insomnia indirectly through PTSD β = −0.04 and depression β = −0.07.	PCEs were significantly associated with fewer insomnia problems regardless of ACEs levels, but the magnitude of protective effect of PCEs was lower for participants with higher ACEs.
Goodyear (2002)	Characteristics of male partner	Author-generated 3-item Likert “Satisfaction with childhood” measure	9.65/21 (4.22)	NR	NR	Satisfaction with childhood not statistically significantly connected with other variables in the model.
Nevarez-Brewster (2022)	Parent sleep quality	BCEs scale (Narayan 2018)	8.9/10 (1.68)	44% of sample endorsed all 10 BCEs	Negative association with sleep problems at the intercept (*b* = −0.91) and throughout pregnancy.	BCEs predicted better sleep quality across gestation even after covarying current life stressors.
Family and Child Outcomes (*n* = 9)						
Barnert (2023)	Offspring arrest before age 26	Childhood Retrospective Circumstances Study (CRCS)	NR	0–1 PCEs 27.9% 2–3 PCEs 50.2% 4–5 PCEs 21.9%	4–5 PCEs associated with 0.53 adjusted OR in model without ACEs	4–5 PCEs associated with reduced offspring arrest, but this effect became statistically insignificant when ACEs added to model.
Bartlett (2015)	Infant neglect and maternal empathy	Parental Bonding Instrument (PBI)	NR	NR	No significant interaction effect of PCEs on the association between childhood neglect and childhood multiple maltreatment on infant neglect.	In a trial of a home visiting program for young mothers, PCEs did not have a statistically significant moderating effect on the relationship between maternal childhood maltreatment/neglect and infant neglect or maternal warmth.
Blackwell (2024)	Child wellbeing and psychopathology	PCEs (Bethell, 2019)	median parent PCEs = 5 IQR: 2, 6	NR	Each additional parent PCE equated to 0.32 increase in child PCEs. An increase in 1 child PCE was associated with 0.10–0.16 increase in well-being and 0.06–0.10 decrease in psychopathology.	Parents’ PCEs were associated with increased PCEs in their offspring. Offspring PCEs were associated with increased well-being and decreased psychopathology.
Daines (2021)	Family functioning	BCEs scale (Narayan, 2018)	8.2/10	NR	Standardized coefficients: family social and emotional health processes 0.24, family healthy lifestyle 0.26, family health resources 0.25, family external social supports 0.31	PCEs were positively associated with each family health domain - family social and emotional health processes, family healthy lifestyle, family health resources, and family external social supports - irrespective of ACEs score.
Gissandaner (2024)	Child externalizing behaviors	BCEs scale (Narayan, 2018)	8.9/10 (1.78)	NR	*r* = −.25 preschool externalizing problems (PEP)	Caregiver PCEs, and not caregiver social-ecological factors, had a significant negative association with PEP. However, this relation became statistically non-significant when adjusting for caregiver ACEs.
Narayan (2019)	Maternal PTSD and depression symptoms, child exposure to traumatic events	Angels in the Nursery Interview (Narayan et al., 2017)	3.04/5 (1.23)	NR	Bivariate correlation −0.17 with maternal stress. β = −0.15 interaction of maltreatment x angel memories predicting PTSD symptoms and maternal comorbid mental health problems. Interaction in maltreatment x angel memories β = 0.17 predicting offspring exposure to traumatic events.	Angel memories significantly moderated associations between parents’ childhood maltreatment and PTSD, comorbid psychopathology, and children’s trauma exposure. Protective effects against children’s trauma exposure were significant only for female children.
Prime (2022)	Family stress and positive adaptation	BCEs (Narayan, 2018)	5.7/7 (1.58)	NR	Bivariate correlations with BCEs: Family stress: −0.10 Pandemic stress: −0.14 Positive adaptation: 0.14 Caregiver distress: −0.26 Caregiver anxiety: −0.17	Overall, those least impacted by pandemic-related disruption reported more caregiver psychosocial resources (including BCEs) and fewer family vulnerabilities than groups with other patterns of pandemic-related disruption.
Reese (2022)	Child adverse family experiences (AFEs)	10-item BCE (Narayan 2018) AND 3 items from PCEs (Bethell 2019)	Women: 11.0/13 Men: 10.9/13	NR	Father’s PCE → Family Health → Child’s AFE Beta = −0.045; Mother’s PCE → Family Health → Child’s AFE Beta = −0.033.	Parental PCEs decreased child’s AFEs indirectly by impacting family health.
Zhu (2023)	Child prosocial behaviors	BCEs (Narayan 2018)	9.1/10 (1.45)	The prevalence rates of each type of PCEs) were BCE 1: 95.3%, BCE 2: 98.1%, BCE 3: 88.8%, BCE 4: 94.7%, BCE 5: 89.9%, BCE 6: 92.3%, BCE 7: 73.1%, BCE 8: 90.6%, BCE 9: 93.7%, and BCE 10: 93.6%	PCEs and total difficulties (OR = 0.88) and prosocial problems (OR = 0.93). When stratified by ACEs scores, low ACEs and high PCEs were associated with a lower risk of offspring total difficulties problems (<4 ACEs: OR = 0.79; ≥ 4 ACEs: OR = 0.89).	PCEs slightly buffered the negative effects of ACEs on offspring’s total difficulties and prosocial problems. Mothers with high PCE scores and higher ACEs were associated with higher risk of offspring total difficulties, while mothers with low ACEs and high PCEs had lower risk of offspring total difficulties.

*aOR* adjusted odds ratio

BCEs key: BCE 1: At least one caregiver with whom you felt safe; BCE 2: At least one good friend; BCE 3: Beliefs that gave you comfort; BCE 4: Enjoyment of school; BCE 5: At least one teacher who cared; BCE 6: Good neighbors; BCE 7: An adult (not a parent/caregiver) who could provide you with support or advice; BCE 8: Opportunities to have a good time; BCE 9: Like yourself or feel comfortable with yourself; BCE 10: Predictable home routine, like regular meals and a regular bedtime

### Quality of Evidence

Appraisal of the evidence revealed that 72% (*n* = 31) of the studies reported approval by an ethics review board. Although these omissions do not necessarily indicate ethics approval was not received, they do indicate that reporting ethics approval was not required by the academic journal. Regarding use of a validated tool to measure PCEs, 72% (*n* = 31) of studies reported using a validated tool, 19% (*n* = 8) reported using items derived from an existing tool, and 7% (*n* = 3) did not use a validated tool or derived items. One study (2%) used only qualitative methods to describe PCEs. The three studies that did not report using a validated or derived tool were published in 2002–2009, before the current wave of PCEs research.

Most (86%, *n* = 37) of the quantitative studies reported using a validated measure of parent or child outcomes, while 5% of studies (*n* = 2) were qualitative and not applicable to this criterion, and 10% (*n* = 4) reported outcome measures with unclear validity. All quantitative studies (*n* = 41) were determined to have used sound statistical methods that were appropriate for the research question. Given that the purpose of scoping reviews is to provide an assessment of the current state of the literature on a topic, rather than to answer a specific clinical question, all studies selected for extraction were included in the results, regardless of their quality.

### Study Designs

Studies used primarily cross-sectional (*n* = 25) or longitudinal cohort (*n* = 13) designs. Other designs included qualitative (*n* = 2), randomized controlled trial (*n* = 2), and quasi-experimental (*n* = 1) designs. Of the studies included, two used qualitative data only, and five used a mixed methods approach.

### Study Settings and Samples

The majority (63%) of studies (*n* = 27) were conducted in the USA. Other studies were conducted in China (*n* = 4), Canada (*n* = 3), Israel (*n* = 2), Portugal (*n* = 1), Ireland (*n* = 1), and Sweden (*n* = 1); four spanned several countries. No studies were conducted in low- or middle-income countries. Most (74%) studies (*n* = 32) were conducted in community settings, outside of clinical or institutional settings. Several studies, particularly those with pregnant women, were clinic-based (*n* = 7) or a combination of community and clinic-based (*n* = 3). One study did not report the setting. Of the 24 studies reporting the geographic classification of their samples, 16 studies were conducted in urban settings, six in urban and rural settings, one in a rural setting, and one in “mostly suburban” communities.

Sample sizes of the quantitative studies ranged from 50 to 7,245 (Median = 262, M = 946, SD = 1,825), with only one nationally representative, U.S.-based sample (Barnert et al., 2023). Ten of the studies (23%) included pregnant individuals, 11 (26%) included mothers only, 17 (40%) included parents of any gender, and four (10%) included heterosexual couples. Mean ages of the study samples ranged from 16 years to 47 years. The majority (*n* = 24) of studies included parents early in their parenting experience (i.e. pregnancy or parenting young children). Only one study enrolled both the parent and child in the sample (Barnert et al., 2023).

Most studies (81%, *n* = 35) reported parents’ race/ethnicity. Of these, 31% (*n* = 11) of the samples were predominantly composed of racial/ethnic minoritized individuals (i.e., Black/African American, Latino, and multiracial individuals); 20% (*n* = 7) were comprised of 80% or more White/Caucasian individuals. Studies conducted in China and Europe did not report the racial/ethnic composition of their samples.

### Measures of PCEs

The quantitative instruments used to measure PCEs are listed in Table 2. A total of eight instruments were used to measure PCEs, and an additional four studies developed their own questionnaires. See Table 3 for all measures and their abbreviations. The most frequently cited instrument (58%; *n* = 23) was the Benevolent Childhood Experiences Scale (BCEs), a 10-item scale that was created as a counterpart to the ACE questionnaire and validated with a diverse sample of pregnant women (Narayan et al., 2018). This scale drew from previous instruments that measured positive childhood experiences, including the Protective and Compensatory Experiences Scale (Morris et al., 2018) and Positive Influences in Childhood (Chung et al., 2008), but aimed to capture a broader range of cultural experiences, particularly of growing up in rural areas and low-middle-income countries. The BCEs scale demonstrated test-retest reliability of 0.80 when administered during prenatal and postpartum periods, and mean levels were comparable across sociodemographic sub-groups (Narayan et al., 2018). Studies have demonstrated the validity of BCEs based on associations with parent stress and mental health (Merrick et al., 2019; Raghunathan et al., 2024).

**Table 3:** PCEs measures’ abbreviations

Abbreviation	Measure Name
BCE scale	Benevolent Childhood Experiences scale
CRCS	Childhood Retrospective Circumstances Study
EMBU	Own Memories of Childhood questionnaire
FOQ	Family of Origin Questionnaire
PACEs survey	Protective and Compensatory Experiences survey
PARQ	Parental Acceptance/Rejection Questionnaire
PCEs questionnaire	Positive Childhood Experiences questionnaire
PBI	Parental Bonding Instrument

The next most frequently cited measure (8%, *n* = 3) was the Positive Childhood Experiences Questionnaire, composed of seven items adapted from the Child and Youth Resilience Measure-28 (CYRM-28; Liebenberg et al., 2013). The Positive Childhood Experiences questionnaire was added to the statewide 2015 Wisconsin Behavioral Risk Survey and was associated with lower depression, fewer mental health problems, and better relational health (Bethell et al., 2019).

Other survey instruments used to measure PCEs were the Protective and Compensatory Experiences (PACEs) survey (Morris et al., 2018), Parental Bonding Instrument (PBI; Parker et al., 1979), the Parental Acceptance/Rejection Questionnaire (PARQ; Rohner et al., 2005), the Family of Origin Questionnaire (FOQ; Lewis & Owen, 1995) and the Own Memories of Childrearing questionnaire (EMBU; Perris et al., 1980). Barnert et al. (2023) used a sub-set of items from the Childhood Retrospective Circumstances Study (CRCS; McGonagle & Freedman, 2015). Four studies used author-generated questionnaires (Chung et al., 2008; Goodyear et al., 2002; Herrenkohl et al., 2013; Suzuki et al., 2009). Narayan et al. (2019; 2017) used a semi-structured, coded interview tool called the Angels in the Nursery Interview to capture memories of feeling “especially loved, understood, or safe.” Two qualitative studies (see Table 4) used primarily semi-structured interviews to gather information about positive childhood experiences and their impact on the individual participants (Greif et al., 2000; Tadjine & Swords, 2025).

**Table 4:** Findings of qualitative studies (*n* = 2)

Authors (Year)	Study Objective(s)	Major Themes Identified	Summary of PCEs-Related Findings
Greif (2000)	“This article, based on interviews with African American mothers of academically successful sons, describes these women’s experiences when they were young in an attempt to learn what family influences affected the parenting that produced academic success.”	• Beliefs received from parents • Messages about education • Observations about gender roles	Mothers of high-achieving young men described upbringings that were rife with messages about the importance of hard work, responsibility, education, and religion. The messages were delivered in an atmosphere in which they felt supported and within which they experienced structure, protection, and accountability.
Tadjine (2025)	“To explore the lived experiences of mothers with a history of ACEs, if they consider their positive childhood experiences when parenting their child, and how they use these positive experiences to break the cycle of intergenerational trauma.”	• Replicating positive experiences • Creating new positive experiences • Protecting children from intergenerational trauma	Participants in this study expressed a strong desire to protect their children from the trauma that they had endured in childhood. They attempted this by replicating those experiences they viewed as positive, and avoiding what they viewed as detrimental factors, including abusive partners and substance abuse.

Instruments to measure PCEs varied in what types of childhood experiences were measured. The PBI, PARQ, and EMBU all captured positive and negative childhood memories and focused on the family environment. The FOQ measured only positive childhood experiences in the family. The CRCS combined items from other PCE measures, including PCEs (Bethell et al., 2019), BCEs (Narayan et al., 2018) and the HOPE framework (Burstein et al., 2021) to measure positive childhood experiences in the family, community, and school settings.

The PACEs, BCEs, and PCEs scales expanded the types of childhood experiences measured. The PACEs survey included five items about relationships, and five items about resources. Both BCEs and PCEs scales captured experiences of feeling safe with a family member, having positive community experiences, and feeling supported by friends and a non-parent caregiver. The BCEs scale also uniquely captured personal experiences, such as beliefs about oneself, while PCEs focused entirely on exposures to household and community experiences. PCEs also expanded into more granular inquiry into family-based experiences by inquiring about family’s support “during tough times” and feeling able to talk to family about feelings. Authors who created their own measures focused on family support and affection (Chung et al., 2008), satisfaction with childhood (Goodyear et al., 2002), family care, warmth, and support (Herrenkohl et al., 2013), and feeling loved and understood by family (Suzuki et al., 2009).

### Models Tested to Understand Intergenerational Impact of PCEs

About half (*n* = 22) of the studies tested a conceptual framework, theory, or model to interrogate the relationships among PCEs, parent or child outcomes, and the nature of the intergenerational transmission of protective factors. The most common frameworks used were resilience theory, angels in the nursery, and ecological systems/eco-developmental models.

The most frequently tested framework was that of “resilience,” a complex phenomenon typically described as the capacity to adapt and function during and/or after adversity (Yule et al., 2019). In the cases included in this review, PCEs were conceptualized as a resilience factor with the potential to promote positive outcomes even amidst adversity. Two studies tested resilience as a mediating variable between PCEs and a dependent variable, parental mental health (Johnson et al., 2022) and child externalizing behaviors (Gissandaner et al., 2024), while other studies applied a resilience framework without directly measuring resilience (Daines et al., 2021; Merrick et al., 2019; Zhan et al., 2021). Two studies analyzed PCEs as a moderator between parents’ experiences of ACEs and their own parenting outcomes (Bartlett & Easterbrooks, 2015; Morris et al., 2021). Finally, Chasson & Taubman—Ben-Ari (2022) examined a concept related to resilience, “personal growth,” as a dependent variable for individuals experiencing pregnancy and the transition to parenthood with varying levels of PCEs.

Several studies by Narayan and colleagues developed and used the concept of “angels in the nursery” to test the intergenerational relationship between parents’ PCEs, maternal mental health, and child’s exposure to adversity (Narayan et al., 2017, 2019, 2020). Arising from clinical observations, the angels in the nursery concept was built upon attachment theory, which posits that experiences in infancy and early childhood form templates for future relational behavior (Narayan et al., 2020). These studies were designed to examine the types and mechanisms of PCEs that impact parents and parenting through recall of those experiences. Taken together, these studies confirmed that parent-reported PCEs were correlated with parents’ reports of angel memories, or “moments when they felt loved, safe, and understood that sustained them through adverse circumstances” (Narayan et al., 2017). These angel memories were found to be protective for parents’ mental health and their child’s exposure to adversity (Narayan et al., 2019, 2020).

Two additional studies used ecosystems/eco-developmental perspectives to inform studies on the interactions of PCEs with other social-environmental factors to promote health-related outcomes, including parent mental health symptoms, parenting stress (Narayan et al., 2018) and attitudes about parenting (Beaton & Doherty, 2007). Other frameworks or theories tested included self-efficacy (Suzuki et al., 2009), attachment (Siddiqui et al., 2000), family systems theory (Reese et al., 2022), the hierarchical taxonomy of psychopathology (HiTOP) model (Clark et al., 2023), and the ACE framework (Chung et al., 2008). Blackwell et al. (2024) generated a conceptual model to understand the relationship between parents’ ACEs and PCEs and children’s outcomes, with children’s ACEs and PCEs as mediating variables.

### Outcomes of PCEs

The majority of studies (67%; *n* = 28) examined parents’ mental health and related outcomes, or parenting beliefs, attitudes, and behaviors. Four studies focused on other parent outcomes, including substance use and sleep problems, and eight studies addressed family and child outcomes, such as family functioning and child behaviors. Summaries of the findings regarding each of these outcomes are described below.

#### Parent Mental Health Outcomes

Among the studies focused on parents’ mental health outcomes, the most frequently examined symptoms were those of post-traumatic stress disorder (PTSD; *n* = 9), depression (*n* = 7), psychological distress (*n* = 3), and anxiety (*n* = 2). One measured post-traumatic growth (Chasson & Taubman-Ben-Ari, 2024), and one measured general psychopathology (Clark et al., 2023). All studies examined symptomatology via self-report survey; none used a complete diagnostic interview or asked about lifetime history of diagnosis. Among studies examining PTSD, depressive symptoms, or psychological distress (*n* = 14), most (*n* = 13) found that PCEs were associated with lower self-reported psychological symptomatology. Among the studies of parents’ mental health symptoms that used linear regression, PCEs’ direct effect sizes were small, ranging from − 0.03 to − 0.35. Several studies used logistic regression, with odds ratios ranging from 0.42 to 0.93 for PCEs effects on psychopathology symptoms. Analyses of PCEs latent classes on outcomes are detailed in Table 2.

#### Parenting Beliefs, Attitudes, and Behaviors Outcomes

PCEs were positively associated with both self-directed and child-directed parenting beliefs and attitudes. PCEs were positively associated with parenting self-efficacy (Chasson & Taubman – Ben-Ari, 2022; Suzuki et al., 2009), parental role satisfaction (Chasson & Taubman – Ben-Ari, 2022; McConnell et al., 2022), “angel memories” (Narayan et al., 2020) and positive attitudes toward nurturing parenting (Morris et al., 2021). PCEs were associated with child-oriented parenting beliefs of prenatal fantasy about the future child (Siddiqui et al., 2000), and satisfaction with child (Simons et al., 1993). Among studies examining PCEs and parenting beliefs, effect sizes were mostly small, ranging from 0.13 to 0.34. Beaton & Doherty (2007) used data from a 3-year RCT with expecting couples to examine the relationship between self-reported closeness with family in childhood and fathers’ attitudes toward fatherhood. In this study, there was no difference between intervention and control groups for attitudes toward fatherhood, so both groups were included in the final analysis. Men had more positive attitudes toward being involved as fathers when their children were infants if they had either very close or distant relationships with their parents during childhood.

Among the studies examining parenting behaviors, PCEs were positively associated with self-reported parenting warmth (McConnell et al., 2022) and negatively associated with self-reported harsh physical discipline (Herrenkohl et al., 2013). Among the studies examining parenting behaviors, statistically significant effect sizes were small, ranging from 0.13 to 0.30. Quality of observed parenting interactions and self-reported maternal response to infant crying were not associated with PCEs (Baptista et al., 2024; Onyskiw et al., 1997). Onyskiw et al. (1997) and Herrenkohl et al. (2013) found differences in parenting beliefs and behaviors differed for mothers compared to fathers. In one quasi-experimental study, Yu et al. (2025) found no relationship between parents’ PCEs and their attendance or engagement in a group-based parenting skills intervention.

While most of the studies of parenting beliefs, attitudes and behaviors examined direct effects, two of the three studies that examined the buffering effects of PCEs on the effects of ACEs found no statistically significant moderation effects (Bailes et al., 2024; Herrenkohl et al., 2013). By contrast, Morris et al. (2021) found that when PCEs were low, ACEs were associated with more favorable attitudes toward harsh parenting. Finally, none of the studies that used observed outcome measures of parenting beliefs or behaviors had statistically significant associations (Bailes et al., 2024; Onyskiw et al., 1997). In one qualitative study, Tadjine & Swords (2025) queried how adverse and positive childhood experiences influenced parenting practices among Irish mothers of young children (see Table 4). This study found that mothers attempted to draw on their positive childhood experiences to inform how they parented their own children, even when they had personal histories of adversity.

#### Other Parent Outcomes

Four studies examined parent outcomes that were not direct indicators of mental health or parenting. PCEs were associated with less insomnia and better sleep quality (Geng et al., 2021; Nevarez-Brewster et al., 2022). One study of marijuana use in lactating individuals found that PCEs did not moderate the association between ACEs and self-reported marijuana use (Crouch et al., 2023). Goodyear et al. (2002) found no relationship between Latina adolescent mothers’ PCEs and the relational traits of their male partners, such as antisocial behavior.

#### Family and Child Outcomes

There were nine studies that reported on the relationship between parents’ PCEs and family and child outcomes, of which seven used parent report of child outcomes, one used adult child self-report (Barnert et al., 2023), and one used substantiated Child Protective Services reports (Bartlett & Easterbrooks, 2015). Two studies examined family-level outcomes. Daines et al. (2021) found that parents’ PCEs were positively associated with four domains of family health (family social and emotional health processes, family healthy lifestyle, family health resources, and family external social supports), irrespective of ACEs. Prime et al. (2022) found that parents’ PCEs were negatively associated with family stress and positively associated with family positive adaptation during the Covid-19 pandemic. Among the six studies examining child outcomes, Blackwell et al. (2024) found that parents’ PCEs were positively associated with the child’s exposure to PCEs, and consequently, child well-being. Parents’ PCEs were negatively associated with child emotional and behavioral difficulties (Blackwell et al., 2024; Gissandaner et al., 2024; Zhu et al., 2023), although in Gissandaner, et al. (2024), those effects did not persist when parents’ ACEs were accounted for. Parents’ PCEs were negatively associated with children’s exposure to traumatic experiences in two studies (Narayan et al., 2019; Reese et al., 2022), but Bartlett & Easterbrooks (2015) found that positive care in childhood did not moderate the relationship between childhood neglect in mothers and the likelihood of engaging in infant neglect themselves.

Two studies included outcomes in adult children. Barnert et al. (2023) found that parents’ PCEs were negatively associated with their adult children’s self-report of arrest before age 26. Among those studies that used linear regression or correlation analyses to examine the relationship between parents’ PCEs and child outcomes, effect sizes were small, ranging from 0.03 to 0.31. In a qualitative study, Greif et al. (2000) queried the role of African American mothers’ positive childhood experiences in raising academically successful sons, finding that beliefs handed down from parents influenced parents and their children (see Table 4).

## Discussion

The purpose of this scoping review was to understand the extent and type of evidence available regarding parents’ PCEs and the impact of parents’ PCEs on their children’s outcomes. Overall, the 43 articles that met inclusion criteria represented a developing field of study with some clear trends and implications for policy and practice, as well as limitations and gaps in knowledge that indicate directions for future research.

### Summary of Findings

Parents’ PCEs were associated with better parent mental health and more positive parenting beliefs and attitudes, while outcomes overall were mixed for parenting behaviors and family and child health outcomes. There was evidence of a clear though modest relationship between parents’ PCEs and better mental health and well-being. This finding aligns with the review conducted by Han et al. (2023), which found that PCEs were associated with improved mental health outcomes. This finding is also an important indicator of PCEs as potential intergenerational protective factor, as parents’ mental health is a significant factor in children’s health (Pierce et al., 2020; Spearman et al., 2023).

Parenting attitudes and beliefs were associated with parents’ PCEs. Parents’ PCEs and their parenting behaviors and practices were less likely to demonstrate significant associations when parenting behavior was observed (e.g. parent-child interactions) rather than parent-reported. Parents’ positive attitudes, beliefs, and practices are all potential protective factors for child wellbeing, given parenting’s central influence on child development (Yamaoka & Bard, 2019). Taken together, the nine studies that included family and/or child outcomes showed promising effects of PCEs for family health and children’s health outcomes. However, in several cases these benefits lacked significance when ACEs were included in the statistical models as covariates, or the benefits were only significant for some demographic sub-groups.

### Strengths and Limitations of the Science and Directions for Future Research

There are some notable strengths in this emerging field of study, as well as several significant gaps that point to directions for future research. These studies represent an increasing awareness of the importance of positive childhood experiences not only for adult health, but for the health of families and future generations. Rigorous examination of these modifiable, health promoting and protective factors is key to discovering points of intervention to improve outcomes for families, interrupt intergenerational transmission of risk, and promote transmission of protective factors.

#### Samples and Settings

Sampling approaches represented both strengths and weaknesses. North American samples reported significant racial and socioeconomic diversity, including populations that have historically been under-represented in clinical research (National Academies of Sciences, Engineering, and Medicine, 2022). Over half of the studies focused on parents who were pregnant or parenting very young children, thereby capturing sensitive periods of early development (Halfon et al., 2014).

However, most samples were relatively small and were comprised primarily of mothers. Non-random, convenience sampling approaches also introduced the likelihood that participants would be predisposed to have certain confounding characteristics and limited generalizability. Additionally, studies with smaller sample sizes may have been underpowered to detect effects or analyze subgroups (i.e., mothers versus fathers). Apart from studies that sampled heterosexual couples together, all of the studies that enrolled both mothers and fathers had samples that were heavily weighted to mothers. With a few studies noting differences in how mothering versus fathering impacted participants as well as how mothers versus fathers were affected by their own PCEs, examination of caregiver gender and caregiver role identities’ influence on the transmission of the benefits of PCEs should be explored.

The context in which children experience PCEs, and in which they later parent their own children, such as adverse or supportive social or economic environments, may have unique influence on PCE prevalence and impact, but at this time that is not well known. With only one nationally representative study included in the United States, much remains unknown about population-level prevalence of parents’ PCEs and outcomes for parents and their children. Additionally, low- and middle-income countries were not represented at all in this review, and there was also a paucity of racially and ethnically diverse samples in Europe. In addition, collectivist cultural contexts were not explicitly represented in any of the articles, which not only represents a gap in the literature, but may also limit interpretations and inferences drawn from BCEs literature as a whole (Hamby et al., 2021). Without this foundational data, public health and clinical initiatives to increase PCEs may be imprecise, ineffective, or not reach populations most at risk.

While some studies on PCEs have addressed unhoused parents and those with intellectual disabilities, research remains limited regarding the prevalence and impact of PCEs among families affected by mental illness (GBD 2019 Mental Disorders Collaborators, 2022) and opioid use disorder in North American adults (Wang et al., 2025). Future research should consider how studies are advancing knowledge of PCEs in diverse or critically underserved populations. Furthermore, many study designs included self-report by only one adult in a family. Therefore, our knowledge of PCEs is hindered not only by mono-method, mono-informant bias, it is also failing to include perspectives of children, fathers, grandparents, and informal caregivers, and observed data, all of which would increase the richness of our understanding of PCEs’ influence not only on individuals but on families and communities.

#### Study Designs

Understanding the intergenerational effects of parents’ PCEs on parenting and children’s outcomes is also limited by several common study design features that introduce potential bias. First, although relying parents’ retrospective reports is an inevitable aspect of PCEs research, recall of past experiences is a known potential source of bias in this research (Reuben et al., 2016). How parents remember their childhoods may be a very important factor in PCEs mechanisms of action, but this remains unknown until we can learn more about actual or observed PCEs compared with how they are recalled in adulthood. Research on retrospective self-report of ACEs suggests that multiple factors may lead to over- or under-estimates of prevalence and impact of PCEs (Coleman et al., 2024). For example, personal history of Major Depressive Disorder or trauma, which are both associated with autobiographical memory impairment, could lead to under-report of positive experiences in childhood (Borrelli et al., 2024; Hallford et al., 2022). In addition to recall bias, parent self-report measures of PCEs and parent-child outcomes may have introduced mono-informant, mono-method bias and contributed to the small statistically significant effects found in these studies. Indeed, studies that used independent observations of parenting or child outcomes found no relationships between PCEs and parent-child outcomes. Perspectives on family and child outcomes were also typically limited by being reported by one parent, rather than a family-wide assessment, including children’s, teachers, or other caregivers’ perspectives.

Another limitation in this body of knowledge is the use of cross-sectional designs, which limit authors’ ability to make causal inferences or determine directionality between the variables. In turn, scientists were more limited in the conclusions they can draw about the causal impact of PCEs, despite results finding relationships between PCEs and health outcomes. There are also missed opportunities to combine traditional PCEs survey data with other reporters’ perspectives or with other observed data sets, such as the child opportunity index (Tyris et al., 2025). Mixed methods and qualitative studies may also help to illuminate how and which types of PCEs had the most impact on their parenting practices.

#### PCEs Measurement

Increased research interest in parents’ PCEs appears to correspond with the publication of several studies describing validated PCEs measures (e.g., Bethell et al., 2019; Narayan et al., 2018). While the use of validated PCE measures aids in replicability and comparison across populations and settings, these scales also typically generate a cumulative score to predict risk. As scholars have noted, PCEs have rarely been examined using dimensional models or typologies, such as the ACEs-oriented Dimensional Model of Adversity and Psychopathology (McLaughlin et al., 2021). There is also insufficient knowledge regarding the timing and chronicity of PCEs in the general population and certainly not in studies focused on parents to our knowledge. Child development theory would suggest that to improve our understanding of intergenerational impacts of PCEs, studies need to better measure how timing, chronicity, and types of experiences may have different effects on children’s future outcomes as adults and, specifically, as parents. More measurement-oriented studies to investigate best practices for measuring and analyzing PCEs in parents is needed.

#### Conceptual Models

Finally, this review revealed a general lack of consensus about *how* PCEs contribute to the health promoting and protective effects that are shown in the literature and a lack of clear frameworks for investigation of the intergenerational transmission of the benefits of PCEs. For example, it would be useful to have working models for understanding how psychosocial environmental factors, potential confounders, influence PCEs and parenting, and subsequent children’s health outcomes. More robust models or frameworks would also assist scientists to identify the potential points of intervention to harness the power of PCEs for children’s health. There is currently a lack of knowledge of interventions that may directly increase the number or potency of PCEs to which children are exposed. While scientists seem to be clear on the benefits of PCEs for parents, approaches to understanding how PCEs influence parenting behavior and next-generation outcomes seem not to be trending in any clear direction at this time.

### Implications for Policy and Practice

There is evidence that PCEs benefit parents’ mental health, an established protective factor for children’s health and wellbeing (Pierce et al., 2020). And, while there are significant gaps in the evidence, several studies in this review point to the potential benefits of parents’ PCEs for children’s wellbeing. Given that PCEs may confer benefits even when ACEs are present, trauma-informed policies focused on reducing ACEs exposure and ameliorating its effects must include interventions that promote PCEs. As the AAP and others have already pointed out, safe, stable, nurturing relationships, an aspect of PCEs, are indeed a biological necessity (Garner & Yogman, 2021). In light of this, policies promoting parents’ mental health care access and providing parents with adequate support to meet their children’s physical and emotional needs is paramount.

While there is a lack of clarity on how different types of PCEs benefit parents, families, and children, the currently validated measures include community-based experiences such as feeling supported by non-parent adults and feeling a sense of belonging in school (Bethell et al., 2019). Policymakers should keep in mind that families raise children in contexts that are influenced by community resources. Community-based interventions may have two ways of supporting the health promotive benefits of PCEs: First, by supporting parents to provide safe, stable, and nurturing environment at home, and second, by creating opportunities for children to experience safety and support outside their homes, such as at school or in neighborhoods. Several community-based interventions, including home visiting programs like the Nurse Family Partnership, and two-generation early childhood programs like Head Start, have experience supporting parents (Bower et al., 2020). Access to these programs should be considered as part of any policy addressing child development, parenting, and social determinants of health. Additionally, policies that promote social cohesion and safe, child-friendly communities may also benefit children and their parents by offering opportunities to feel safe and supported. Examples of such programs include the provision of safe, social activities at community centers and libraries, and intergenerational programming for seniors and young children at centers like the YMCA or in schools.

Clinicians should consider ways that they can assess for and promote PCEs across populations using validated measures and evidence-based interventions. Pediatric primary care providers are optimally positioned to assess and promote PCEs by establishing trusting relationships with the parents and families with whom they work and supporting them in providing safe and nurturing experiences for their children. For example, Reach Out and Read provides resources at primary care visits for parents wishing to read with their children (Reach Out and Read, 2025), while Healthy Steps, also embedded in primary care settings, provides specialized child development support to parents (Zero to Three, 2025). Finally, community health providers working with children and parents, especially school nurses, can educate their communities on the benefits of PCEs and aim to assess and promote PCEs at community and individual levels as key determinants of health.

### Limitations of this Review

This review has several limitations. First, articles were limited to English language based on the authors’ shared language skill set. This may have led to the exclusion of relevant non-English articles, potentially introducing bias related to cultural or socioeconomic contexts in PCEs research. Second, because no single, universally accepted term exists for positive childhood experiences, our search strategy included a number of terms used to describe PCEs (see Appendix A). Nevertheless, it is possible that some studies using alternative terminology were not captured. Indexing of PCEs in article databases and broader consensus about terms used to describe PCEs would strengthen future systematic reviews. Finally, our search was limited to populations representing parents and primary, non-institutional caregivers, potentially missing literature on PCEs in other caregivers, such as extended family members and childcare providers.

## Conclusion

PCEs have been shown to be an independent predictor of healthy outcomes even when ACEs are present, a finding confirmed by this review of the literature on parents’ PCEs. As a result, interventions to promote PCEs in families and communities should not wait. However, while there are promising findings, PCEs’ influence on next-generation outcomes have not been thoroughly investigated. Further research incorporating longitudinal and/or multiple data sources, as well as more direct examination of the underlying mechanisms of PCEs on parenting and children’s health outcomes is needed. This is an area of research that has the potential to contribute to improving health across populations and merits significantly more and higher quality research to refine and deliver useful interventions for those parents most in need of support.

## Supplementary Information

Below is the link to the electronic supplementary material.

ESM 1 Supplementary Material 1 (DOCX 9.57 KB)

## Declarations

### Public Relevance

This scoping review examines the current scientific literature to understand the extent and type of evidence available regarding parents’ positive childhood experiences (PCEs) and the potential impact of parents’ PCEs on intergenerational transmission of PCEs and protective factors for children’s health and psychosocial outcomes.
