# Supplementary material for: Parents’ Positive Childhood Experiences: A Scoping Review
Source: Advers Resil Sci. 2026 May 23;7(3):34. doi: 10.1007/s42844-026-00219-1 (PMC13198480; doi:10.1007/s42844-026-00219-1)
Supplement: Supplementary file 1 — Supplementary Material 1 (DOCX 9.57 KB) [file 42844_2026_219_MOESM1_ESM.docx]

Appendix A - Search Strategy by Database

Databases (N = 6): PubMed, PsycInfo, CINAHL, Scopus, Embase, Sociological Abstracts

PubMed

"positive childhood experiences" [tiab:~3] OR "benevolent childhood experiences" [tiab:~3] OR "counter ACEs" [tiab:~3] OR "safe stable nurturing relationships" [tiab:~4] OR "positive childhood experience" [tiab:~3] OR "benevolent childhood experience" [tiab:~3] OR "advantageous childhood experience" [tiab:~3] OR "advantageous childhood experiences" [tiab:~3] OR "counter ace" [tiab:~3]

PsycInfo

TX ((benevolent OR positive OR advantageous) N3 ("childhood experience*")) OR (counter N3 (ace OR aces)) OR (safe N2 stable N2 "nurturing relationship*)

CINAHL

TX ((benevolent OR positive OR advantageous) N3 ("childhood experience*")) OR (counter N3 (ace OR aces)) OR (safe N2 stable N2 "nurturing relationship*)

Scopus

TITLE-ABS-KEY ( ( benevolent W/3 "childhood experience*" ) OR ( positive W/3 "childhood experience*" ) OR ( advantageous W/3 "childhood experience*" ) OR ( counter W/3 ace ) OR ( counter W/3 aces ) OR ( counter-ace OR counter-aces ) OR ( safe W/2 "nurturing relationship*" ) OR ( stable W/2 "nurturing relationship*" ) )

Embase

(((benevolent OR positive OR advantageous) NEAR/3 'childhood experience*'):ti,ab,kw) OR ((counter NEAR/3 (ace OR aces)):ti,ab,kw) OR ((safe NEAR/2 'nurturing relationship*'):ti,ab,kw) OR ((stable NEAR/2 'nurturing relationship*'):ti,ab,kw)

Sociological Abstracts

noft((((benevolent OR positive OR advantageous) NEAR/3 'childhood experience*')) OR ((counter NEAR/3 (ace OR aces))) OR ((safe NEAR/2 'nurturing relationship*')) OR ((stable NEAR/2 'nurturing relationship*')))
